# Aortic root translocation (Nikaidoh) procedure for complex transposition of the great arteries with left ventricular outflow tract obstruction

**DOI:** 10.1016/j.xjtc.2023.10.006

**Published:** 2023-10-18

**Authors:** Emile A. Bacha, Stephanie N. Nguyen, Rachel Vanderlaan, Damian J. LaPar, Andrew B. Goldstone, David M. Kalfa

**Affiliations:** Section of Pediatric and Congenital Cardiac Surgery, New York Presbyterian-Morgan Stanley Children’s Hospital, Columbia University Irving Medical Center, New York, NY

**Keywords:** Nikaidoh operation, aortic root translocation, transposition of the great arteries, biventricular repair

## Abstract

**Background:**

Several surgical techniques have been developed for the management of complex transposition of the great arteries with ventricular septal defect and left ventricular outflow tract obstruction (TGA/VSD/LVOTO). Aortic root translocation, or the Nikaidoh operation, offers the most anatomic biventricular repair in these patients. However, the Nikaidoh operation commonly has been limited to patients with “typical” anatomy, including a conoventricular VSD and usual coronary anatomy. We sought to describe a single surgeon’s experience with aortic root translocation for complex TGA/VSD/LVOTO.

**Methods:**

We present a series of 12 patients with complex anatomy who underwent the Nikaidoh operation over the last 13 years.

**Results:**

We report good mid- to long-term results, excellent performance of the reconstructed left ventricular outflow tract, aortic valve competence, and no coronary insufficiency.

**Conclusions:**

Our experience suggests that the Nikaidoh operation is a valid option even for patients with complex TGA/VSD/LVOTO.

**Figure:**
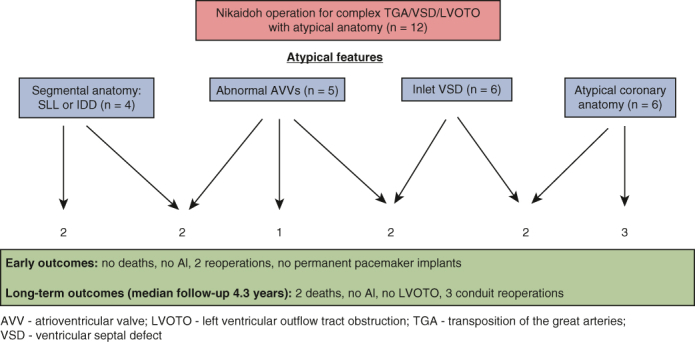
En bloc aortic root translocation with the coronary arteries attached.

**Central Message**

Even with the variable and challenging anatomy often seen in complex forms of TGA/VSD/LVOTO, the Nikaidoh operation may be performed with excellent results in this subset of patients.

**Perspective**

Our series adds to the few isolated reports on aortic root translocation for complex forms of TGA/VSD/LVOTO. Given its excellent durability and long-term outcomes, the Nikaidoh operation, despite its high degree of technical complexity, should be considered for selected patients with unfavorable anatomy for a Rastelli procedure, particularly at experienced centers.

At many experienced centers, patients with classic forms of d-looped transposition of the great arteries (d-TGA) with ventricular septal defect (VSD), left ventricular outflow tract obstruction (LVOTO), and the usual coronary anatomy are managed with an aortic root translocation procedure known as the Nikaidoh operation.^1^ The Rastelli operation, with its long and angulated subaortic baffle, is now reserved for highly selected patients. Surgical management of complex or atypical forms of TGA/VSD/LVOTO remains a challenge owing to wide anatomic variability in the arrangement of the great arteries; size and location of the VSD, the atrioventricular valve (AVV), and its subvalvular apparatus; and coronary anatomy. With increasing experience with the Nikaidoh operation, some surgeons have extended its use to complex forms of TGA/VSD/LVOTO,^2^, ^3^, ^4^, ^5^, ^6^, ^7^, ^8^ which may involve the following anatomic variants: congenitally corrected transposition of the great arteries (cc-TGA), a remote inlet or complete atrioventricular canal-type VSD with no outlet extension, unusual coronary patterns, or straddling AVVs. Here we present a single surgeon’s experience (E.A.B.) with aortic root translocation for complex TGA/VSD/LVOTO over the last 13 years.

## Methods

### Patient Cohort

Between January 2010 and February 2023, a total of 16 patients with a diagnosis of TGA or double-outlet right ventricle (RV) with VSD and LVOTO underwent an aortic root translocation by a single surgeon (E.A.B.) at our institution. Of those, 12 had complex anatomy and were included in the present series. The decision to perform the Nikaidoh operation instead of a Rastelli-type repair was at the discretion of the surgeon and was based mainly on the conal septal anatomy, coronary pattern, and mechanism of LVOTO.

This study was approved by our hospital’s Institutional Review Board (approval AAAU5073, January 12, 2023), with a waiver of individual consent.

### Operative Technique

A median sternotomy is performed, and cardiopulmonary bypass is established with bicaval cannulation and moderate hypothermia. Any prior palliative shunts and the ligamentum arteriosum are ligated and divided. The great vessels are dissected, and the branch pulmonary arteries are extensively mobilized to the hilum. The coronary arteries are mobilized circumferentially as far distally as possible to allow for en bloc translocation of the aortic root with the coronary arteries attached. We do not reimplant the coronary buttons, as we strongly believe that nonanatomic reimplantation results in a higher incidence of aortic regurgitation.

Following aortic cross-clamping and cold cardioplegic cardiac arrest, the right atrium is opened, and the intracardiac anatomy is inspected. The ascending aorta is transected at the level of the pulmonary artery (PA) bifurcation, and the main PA is transected just above the sinotubular junction. We assess the location of the VSD and conal septum, the size of the pulmonary annulus, and AVV morphology to verify the appropriate anatomy for a Nikaidoh procedure.

The aortic root is then harvested from the RV (with the coronaries left in situ), starting a few millimeters below the aortic annulus on the right ventricular outflow tract (RVOT) and continuing circumferentially below the valve, leaving a muscular rim of 3 to 5 mm. The conal septum is then transected down into the VSD, thereby relieving the LVOTO. Any chordal attachments to the septum are carefully noted and kept on their correct side; chordae that are straddling the septum are detached and later reattached to the VSD patch.

The aortic root is translocated posteriorly ensuring no tension or kinking of the coronary arteries and sutured to the neo-LVOT using interrupted, mattressed polypropylene sutures. The posterior two-thirds of the aortic root are sutured to the rim of pulmonary annulus and the cut edge of the conal septum. Anteriorly, the LVOT is reconstructed by closure of the VSD with a patch extending from the crest to the aortic neo-annulus. The patch should protrude beyond the free edge of the aortic root, as it later can serve as the floor of the RVOT if a nonconduit RVOT reconstruction is planned. Any transected chordal attachments are reattached to the patch. The remaining one-third of the aortic root is then anchored to the free edge of the VSD patch to complete the LVOT reconstruction. A Lecompte maneuver is performed, and the ascending aorta is reanastomosed—often after shortening it, to avoid a posterior bulge and compression of the PA—and the RVOT is reconstructed with an orthotopic conduit or as a transannular patch with the cross-clamp applied. For these long cases, we prefer to have a competent pulmonary valve present as opposed to free pulmonary regurgitation and hence lean toward using a conduit. In many cases, the native RVOT opening is very wide and crescent-shaped; these corners can be troublesome bleeding spots and thus are closed primarily to create a more circular orifice. Video 1 demonstrates intraoperative footage of the Nikaidoh operation for complex TGA/VSD/LVOTO.

## Results

### Preoperative Characteristics

The median age and weight at surgery was 2.5 years (range, 0.3-13.0 years) and 14.5 kg (range, 5.8-45.0 kg), respectively. The great vessel relationship was d-TGA in 9 patients (75.0%), of whom 4 (44.4%) had DORV and 3 (25.0%) had cc-TGA. One patient (8.3%) had situs inversus. All patients had severe LVOTO at the time of surgery, which ranged from valvar pulmonary stenosis to tunnel-like LVOTO with pulmonary atresia. Six patients (50.0%) had a remote inlet VSD with no outlet extension. Atypical coronary anatomy was present in 5 patients (41.7%), including inverted coronaries with the right coronary artery arising from sinus 1 and crossing the RVOT (n = 2), dual left anterior descending arteries (n = 1), circumflex artery arising from the right coronary artery (n = 1), and a large conal branch arising from a separate ostium in sinus 2 (n = 1).

Seven patients (58.3%) had at least 1 abnormal AVV, which included a straddling right AVV in 2 patients and moderate right AVV hypoplasia and stenosis, a right AVV with important chordal attachments to the conal septum, a left AVV with important chordal attachments to the VSD crest, a common AVV with a large cleft, and fibrous continuity of both AVVs with the aortic valve in 1 patient each. Nine patients (75.0%) had undergone a prior palliative procedure, including a bidirectional Glenn in 6, Blalock-Taussig-Thomas shunt in 5, balloon atrial septostomy in 2, and PA band in 2. Patients’ anatomic and clinical data are presented in Table 1.

**Table 1:** Summary of patient anatomic and operative data

Patient	Age, y	Weight, kg	Primary diagnosis	Segmental anatomy	VSD type	AVV morphology	Coronary anatomy	Palliative procedures	Anatomic repair	RVOT reconstruction	Rhythm at discharge
1	1.7	10	DORV/d-TGA, VSD, LVOTO	{S,D,D}	Conoventricular	Both AVVs in fibrous continuity with aortic valve	1LR-2Cx (inverted), RCA crossing RVOT	BAS, BTT shunt	Aortic translocation	18 mm aortic homograft	NSR
2	0.4	8	d-TGA, VSD, LVOTO	{S,D,D}	Inlet	Straddling RAVV	1LCx-2R (usual)	BAS, BTT shunt	Aortic translocation	16 mm Contegra	Intermittent first-degree AV block
3	2.3	11	d-TGA, VSD, LVOTO	{S,D,D}	Conoventricular	Normal	1LR-2Cx (inverted), RCA crossing RVOT	None	Aortic translocation, primary VSD closure with aortic root muscle skirt	TAP with CorMatrix patch	NSR
4	1.1	7	DORV/d-TGA, VSD, LVOTO	{S,D,D}	Inlet	Straddling RAVV	1LCx-2R (usual)	BTT shunt	Aortic translocation, RAVV chordal reimplantation	TAP with Contegra patch	NSR
5	2.2	12	Situs inversus, DORV/d-TGA, AVSD, LVOTO	{I,D,D}	Inlet	Common AVV with cleft	1LCx-2R (usual)	BDG	Aortic translocation, AVSD repair, atrial baffle of hepatic veins, LPA plasty, BDG takedown	16 mm Contegra	NSR
6	4.2	18	cc-TGA, VSD, LVOTO	{S,L,L}	Inlet	LAVV chordal attachments to VSD crest	1R-2LCx (usual)	BTT shunt, BDG	Aortic translocation, hemi-Mustard	15 mm pulmonary homograft	NSR
7	8.3	35	cc-TGA, VSD, LVOTO	{S,L,L}	Conoventricular	Normal	1R-2LCx (usual)	None	Aortic translocation, Senning	18 mm Contegra	NSR
8	0.3	5.8	Mesocardia, cc-TGA, VSD, LVOTO	{S,L,L}	Conoventricular	Normal	1R-2LCx (usual)	None	Aortic translocation, primary VSD closure with aortic root muscle skirt, Senning, PPM insertion (preoperative complete heart block)	14 mm pulmonary homograft	Paced
9	2.7	17.0	DORV/d-TGA, VSD, tunnel-like LVOTO with pulmonary atresia	{S,D,D}	Inlet	Normal	1LCx-2R with separate moderate RCA conal branch	BDG	Aortic translocation, VSD enlargement, LPA plasty	16 mm Contegra	NSR
10	8.0	30	d-TGA, VSD, LVOTO	{S,D,D}	Inlet	RAVV chordal attachments to conus	1L-2RCx (anomalous)	PAB, BDG	Aortic translocation, branch PA plasty, BDG and PAB takedown	16 mm Contegra	NSR
11	13.0	45	d-TGA, VSD, LVOTO	{S,D,D}	Conoventricular	Normal	1LCx-2R (dual LAD)	PAB, BDG	Aortic translocation, primary VSD closure with aortic root muscle skirt, branch PA plasty, BDG and PAB takedown	16 mm Contegra	NSR
12	2.9	17.5	d-TGA, VSD, LVOTO	{S,D,D}	Conoventricular	Moderate RAVV hypoplasia and stenosis	1LCx-2R (usual)	BTT shunt, BDG	Half-Nikaidoh, LPA plasty	15 mm pulmonary homograft	Junctional (present preoperatively)

*VSD*, Ventricular septal defect; *AVV*, atrioventricular valve; *RVOT*, right ventricular outflow tract; *DORV*, double-outlet right ventricle; *d-TGA*, dextro-transposition of the great arteries; *LVOTO*, left ventricular outflow tract obstruction; *NSR*, normal sinus rhythm; *RAVV*, right atrioventricular valve; *BAS*, balloon atrial septostomy; *BTT*, Blalock-Taussig-Thomas; *AV*, atrioventricular; *RCA*, right coronary artery; *TAP*, transannular patch; *TGA*, transposition of the great arteries; *AVSD*, atrioventricular septal defect; *BDG*, bidirectional Glenn; *LAVV*, left atrioventricular valve; *cc-TGA*, congenitally corrected transposition of the great arteries; *PPM*, permanent pacemaker; *LPA*, left pulmonary artery; *PAB*, pulmonary artery band; *PA*, pulmonary artery.

### Early Surgical Outcomes

There were no operative mortalities. The median cardiopulmonary bypass time was 193 minutes (range, 166-377 minutes), and the median cross-clamp time was 125 minutes (range, 101-184 minutes). Two patients underwent early reoperations. Patient 8, the smallest patient in the series at 5.8 kg, underwent reoperation on postoperative day 1 for iatrogenic aortic insufficiency (AI) owing to a malpositioned annular suture causing distortion of the posterior aortic valve cusp; removing the suture restored aortic valve competency. Patient 9 had a complex postoperative course complicated by VSD patch dehiscence, recurrent subpulmonic outflow obstruction, and a recalcitrant chylothorax necessitating VSD patch revision, RVOT plasty, and takedown of the superior cavopulmonary connection 2 months later. He recovered well from the procedure, but was returned to the operating room 3 weeks later for repair of an RVOT pseudoaneurysm. Patient 11 had low cardiac output early due to massive aortopulmonary collaterals and required coiling on postoperative day 4.

No patient required an unplanned permanent pacemaker. Patient 8 developed de novo high-grade atrioventricular block preoperatively and underwent concomitant epicardial pacemaker insertion. Patients 2 and 12 were discharged with intermittent first-degree atrioventricular block and junctional rhythm (also present preoperatively), respectively. The remaining patients were in normal sinus rhythm at discharge.

The median postoperative length of stay was 9.5 days (range, 6.0-83.0 days). Except for 1 patient with moderate left ventricular dysfunction (patient 9), all had normal biventricular function, no hemodynamically significant outflow tract obstruction, and no more than mild AI at discharge.

### Late Follow-up

At a median follow-up of 4.3 years (range, 0.3-12.3 years), there were 2 late deaths (Table 2). Patient 5 had heterotaxy syndrome with asplenia and died 12 months postoperatively from infectious causes. Patient 9 died 6 months after his last surgery following a 2-week history of viral illness and emesis that resulted in severe metabolic derangements and ultimately in cardiac arrest at home. In both patients, the latest echocardiogram showed an excellent repair with no more than mild systolic dysfunction and no AI or residual outflow tract obstruction.

**Table 2:** Summary of patient follow-up and echocardiographic data

Patient	Last known clinical status	Reinterventions			Latest echocardiography findings				Follow-up, y
		Procedure	Interval, y	Indication	AI	LVOTO	RV-PA conduit	Ventricular dysfunction	
1	Alive	Conduit balloon angioplasty Conduit replacement	4.0 9.3	Distal conduit obstruction Conduit stenosis	None	None	No PS No PR	None	9.3
2	Alive	Transcatheter PVR PVR, TVr	9.7 9.7	Conduit stenosis/regurgitation Iatrogenic transection of tricuspid papillary muscle during TPVR	None	None	No PS Mild PR	None	12.3
3	Alive	None	-	-	None	None	-	None	11.0
4	Alive	None	-	-	None	None	No PS Free PR	None	5.7
5	Deceased (infection)	None	-	-	Mild	None	Mild PS No PR	None	1.0
6	Alive	None	-	-	Mild	None	Mild PS No PR	None	8.0
7	Alive	Conduit balloon angioplasty, LPA/RPA stent Conduit and RPA stent	1.9 4.7	Distal conduit stenosis and hypoplastic PAs Distal conduit and proximal RPA stenosis	None	None	No PS Moderate PR	None	7.3
8	Alive	AVr	POD 1	Severe AI	Mild	None	Moderate PS Mild PR	None	2.9
9	Deceased (hypoglycemia)	VSD repair, RVOT plasty, BDG takedown Redo RVOT plasty	0.2 0.2	VSD patch dehiscence, RVOTO, recalcitrant chylothorax Pseudoaneurysm of RVOT patch	None	None	Moderate PS No PR	Moderately reduced global LV function	0.4
10	Alive	None	-	-	None	None	No PS No PR	None	0.7
11	Alive	Coiling of aortopulmonary collaterals	Early postoperative	Heart failure	None	None	No PS No PR	None	0.7
12	Alive	None	-	-	None	Mild	No PS No PR	None	0.3

*AI*, Aortic insufficiency; *LVOTO*, left ventricular outflow tract obstruction; *RV*, right ventricular; *PA*, pulmonary artery; *PVR*, pulmonary valve replacement; *TVr*, tricuspid valve repair; *TPVR*, transcatheter pulmonary valve replacement; *PS*, pulmonary stenosis; *PR*, pulmonary regurgitation; *LPA*, left pulmonary artery; *RPA*, right pulmonary artery; *AVr*, aortic valve repair; *POD*, postoperative day; *VSD*, ventricular septal defect; *RVOT*, right ventricular outflow tract; *LV*, left ventricle.

Two patients underwent late surgical reintervention. Patient 1 required balloon angioplasty of the distal conduit 4.0 years later and conduit replacement 9.3 years later. Patient 2 developed conduit failure and underwent transcatheter pulmonary valve implantation 9.7 years later, which was complicated by transection of a tricuspid papillary muscle and severe tricuspid regurgitation. The patient subsequently underwent open pulmonary valve replacement and tricuspid valve repair the next day. Patient 7 underwent a conduit balloon angioplasty and bilateral branch PA stenting 1.9 years later, followed by stent placement within the distal conduit and proximal right PA 4.7 years after that. At the time of this report, no patients had more than mild LVOTO or mild AI at the latest follow-up. Patient 2 remained in intermittent first-degree atrioventricular block, and the remaining patients were in normal sinus rhythm. All survivors are currently New York Heart Association class I.

## Discussion

The Nikaidoh procedure was initially developed for standard forms of d-TGA with a conoventricular VSD and LVOTO. Despite offering the advantages of a more anatomic repair, the Nikaidoh procedure involves a higher degree of technical complexity owing to manipulation of the aortic root. Consequently, the risks of iatrogenic aortic valve incompetence and coronary ischemia are significantly higher than with a Rastelli operation. In complex forms of TGA/VSD/LVOTO, such as those involving unusual coronaries or a remote VSD, surgeons may revert to using a Rastelli-type operation or even a single ventricle approach. Although the Nikaidoh is particularly challenging in this setting, our series demonstrates that aortic translocation can be safely extended to this group of patients with excellent durability and long-term outcomes. This subset of patients presents a unique set of technical considerations, as discussed in detail below.

### Anatomic Biventricular Outflow Tract Reconstruction and Management of Inlet VSDs

At several centers, the Nikaidoh is now the operation of choice for patients with d-TGA/VSD/LVOTO. Benefits over the Rastelli operation include a more natural, direct alignment of the LVOT and RVOT and excellent exposure of the outflow tracts, especially after transection of the conus.^8^ Moreover, because the aortic root is moved posteriorly, RV–PA continuity may be established at the level of the explanted aortic root (ie, the natural RVOT orifice), away from the sternum, thereby avoiding compression and facilitating future reentry. Early concerns regarding AI have been largely resolved by avoiding coronary button takedown and reimplantation, as well as with a very precise geometric root transfer.^7^ Therefore, we generally prefer aortic root translocation over double-root translocation where the coronaries must be taken down and reimplanted by necessity.

Aortic translocation is particularly advantageous in the setting of an inlet or remote VSD, where the Rastelli either carries a great risk of LVOTO due to the curvaceous tunnel or is simply impossible. By dividing and resecting the conal septum, a straight LVOT can be achieved. In some cases, it is possible to avoid using a VSD patch completely and reconstruct the outlet septum with the muscular cuff, as was done in 2 cases in this series. Inlet VSDs can be managed similarly by approaching them from above, connecting the aortic annular opening (after enucleation of the aortic root) with the VSD, and leaving all tricuspid valve attachments on the right side. This generally has been sufficient to create a sufficient LVOT without formal enlargement of the VSD, because the conal division is then closed by the VSD patch.

### Management of Complex Coronary Artery Patterns

Unusual coronary anatomy (ie, atypical coronaries for d-TGA) was previously considered a relative contraindication to the Nikaidoh procedure.^8^ With more experience, we now routinely harvest the aortic root en bloc and leave the coronary arteries in situ, as we have found that the harvest and reimplantation of coronary buttons tends to distort aortic root geometry and is likely the cause of neo-AI. Our single instance of early postoperative AI was due to a technical error. An annular suture had distorted the aortic valve, and removal of the offending suture resolved the issue.

When translocating the root en bloc, the key is to mobilize the coronary arteries circumferentially far enough out on the myocardium. In this series, 2 patients had inverted coronary arteries (1LR-2Cx), and the anterior and posterior loops were managed with extensive circumferential dissection to avoid kinking or stretching of the vessels. In both cases, the right coronary artery crossed over the RVOT. Because RV–PA continuity is constructed in the native RVOT, the crossing coronary remains anterior to the RVOT and not underneath a heterotopic conduit as in a Rastelli. Therefore, coronary compression is not an issue with the Nikaidoh procedure as it is with the Rastelli operation. In fact, the initial plan in 1 of these patients (patient 3) was to perform a Rastelli operation; however, we pivoted intraoperatively to an aortic translocation procedure on visualization of the right coronary artery crossing the RVOT, as this technique avoids division of the RVOT conus.

### Management of cc-TGA

Three patients in our cohort had cc-TGA, including 2 with {S,L,L} segmental anatomy and 1 with situs inversus {I,D,D} segmental anatomy. All had usual coronaries for their anatomy. In these patients, the operation does not vary much from aortic translocation for d-TGA/VSD/LVOTO, except that the plane of the translocation is not anterior/posterior but rather more oblique, in a right/left transverse orientation, as the great arteries are often side-by-side or obliquely related. Given that the atrial switch is rarely performed today, we prefer to wait until these patients weigh at least 8 to 10 kg. In {S,L,L} patients in particular, the conduction system is at risk and often runs anterior and superior to the pulmonary valve before descending along the anterior margin of the VSD. Therefore, division of the conus should occur as leftward as safely possible, and care should be taken when suturing the VSD patch to avoid injury to conduction tissue. No patients in our series developed postoperative complete atrioventricular block necessitating a permanent pacemaker.

### Limitations

Limitations of this series include its retrospective nature and potential lack of external validity given the small cohort size and single-surgeon experience. Additionally, given the rarity and anatomic heterogeneity of this lesion, a multicenter collaboration with long-term follow-up is needed to evaluate the durability and efficacy of the Nikaidoh operation for complex TGA/VSD/LVOTO.

## Conclusions

Overall, the present series adds to the few isolated reports on aortic translocation and biventricular outflow tract reconstruction for complex or atypical forms of TGA/VSD/LVOTO. In experienced centers, the Nikaidoh operation is a particularly valuable option for patients with unfavorable anatomy for a Rastelli procedure. We demonstrate that the benefits of the Nikaidoh operation—the most anatomic biventricular repair possible—may be extended to these patients with variable and challenging anatomy, with excellent results.

## Conflict of Interest Statement

The authors reported no conflicts of interest.

The *Journal* policy requires editors and reviewers to disclose conflicts of interest and to decline handling or reviewing manuscripts for which they may have a conflict of interest. The editors and reviewers of this article have no conflicts of interest.

## Supplementary Data

Video 1 Intraoperative video of a Nikaidoh operation performed for complex transposition of the great arteries with left ventricular outflow tract obstruction. Video available at: https://www.jtcvs.org/article/S2666-2507(23)00386-3/fulltext.
