# Supplementary figures and images for: Aortic root translocation (Nikaidoh) procedure for complex transposition of the great arteries with left ventricular outflow tract obstruction
Source: JTCVS Tech. 2023 Oct 18;22:243–50. doi: 10.1016/j.xjtc.2023.10.006 (PMC10750979; doi:10.1016/j.xjtc.2023.10.006)

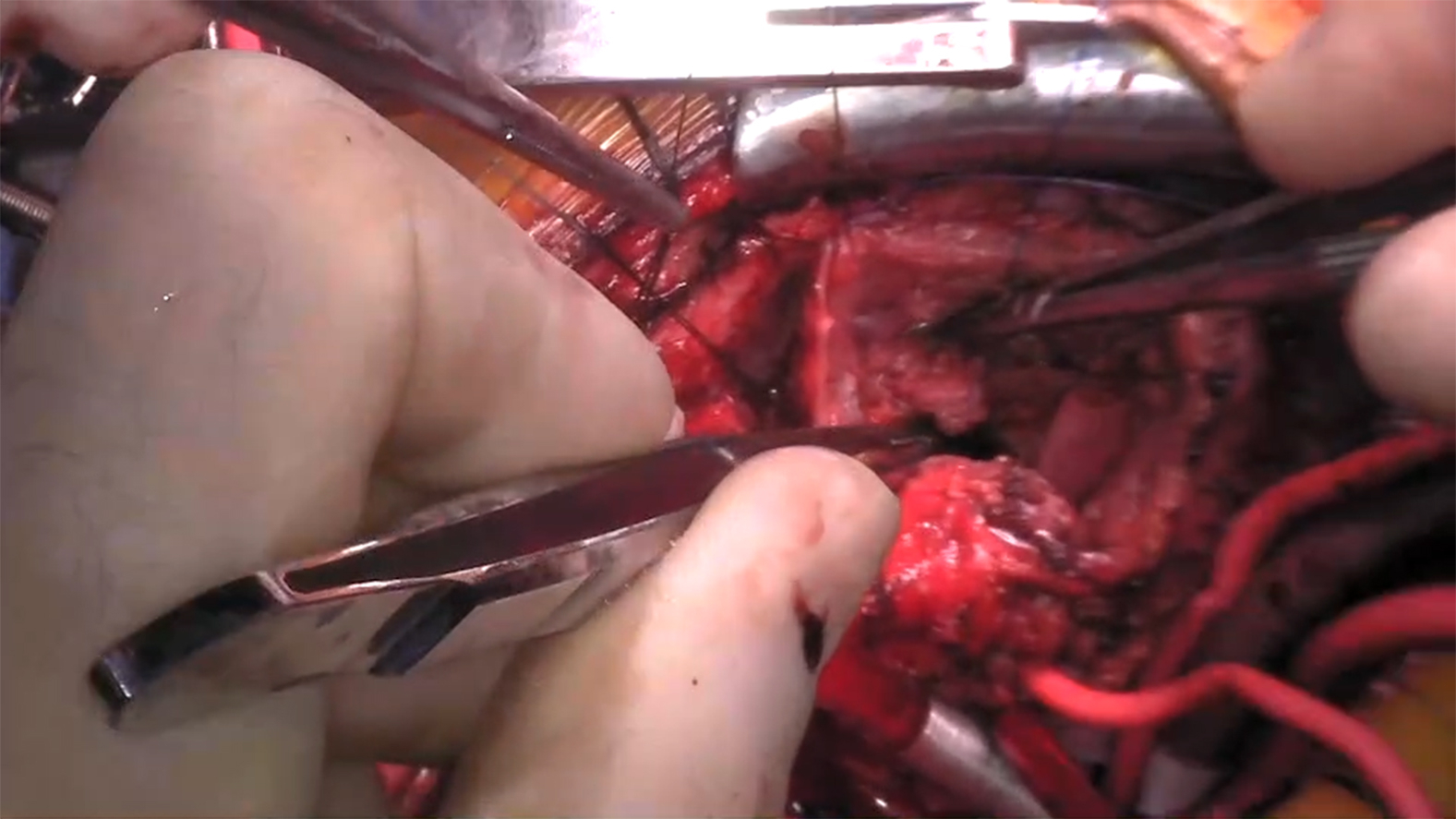

Supplement: Video 1 — Intraoperative video of a Nikaidoh operation performed for complex transposition of the great arteries with left ventricular outflow tract obstruction. Video available at: https://www.jtcvs.org/article/S2666-2507(23)00386-3/fulltext. [file fx2.jpg]
